# Role of carotid duplex imaging in carotid screening programmes – an overview

**DOI:** 10.1186/1476-7120-6-34

**Published:** 2008-07-04

**Authors:** Muhammad A Saleem, Umar Sadat, Stewart R Walsh, Victoria E Young, Jonathan H Gillard, David G Cooper, Michael E Gaunt

**Affiliations:** 1 Cambridge Vascular Unit, Addenbrooke's Hospital, Cambridge University Hospitals NHS Foundation Trust, Cambridge, UK; 2 University Department of Surgery, Cambridge University Hospitals NHS Foundation Trust, Cambridge, UK; 3 University Department of Radiology, Cambridge University Hospitals NHS Foundation Trust, Cambridge, UK

## Abstract

**Background:**

Stroke is the third most common cause of death in the UK and the largest single cause of severe disability. Each year more than 110,000 people in England suffer from a stroke which costs the National Health Service (NHS) over GBP2.8 billion. Thus, it is imperative that patients at risk be screened for underlying carotid artery atherosclerosis.

**Aim:**

To assess the role of carotid ultrasound in different carotid screening programmes.

**Methods:**

A literature overview was carried out by using PubMed search engine, to identify different carotid screening programmes that had used ultrasound scan as a screening tool.

**Results:**

It appears that the carotid ultrasound is an effective method for screening carotid artery disease in community as it effectively predicts the presence of stenosis with high accuracy. There is a need for primary care to recommend high risk patients for regular screening, to reduce stroke and transient ischemic attack (TIA) related morbidity and mortality.

**Conclusion:**

Screening programmes using carotid ultrasonography contribute to public health awareness and promotion which in long term could potentially benefit in disease prevention and essentially promote better standards of healthcare.

## Background

Stroke is the most common cause of severe disability in the UK. Each year more than 110,000 people in England suffer from a stroke which costs the NHS over £2.8 billion. Community-based vascular screening programs play a key role in early vascular disease detection and accurate diagnosis can potentially be cost-effective and in lonXger term, promote health and increase life expectancy.

The main purpose to diagnose carotid artery disease is prevention of stroke in a high risk population. Common factors have been identified in multiple studies that successively characterize the risk of developing carotid artery stenosis in a selected population of patients. In a study by Jacobowitz et al. (2003) [1] the risk factors evaluated included smoking, hypertension, cardiac disease or hypercholesterolemia where altogether, increases the chances of developing cardiac stenosis by ≥ 50%. The absence of all these risk factors would reduce the incidence of cardiac stenosis to 1.8%. Consecutively, each risk factor would increase the chances of developing the incidence of disease by 5.8%, 13.5%, 16.7% and 66.7%[1]. Thus, statistically the probability of cardiac stenosis significantly increases with presence of risk factors. This would further isolate the group of patients that would benefit most, from this particular screening programme.

## Methods

A Pub Med search was performed using keywords "carotid", "carotid artery disease", "carotid artery stenosis", "duplex", "ultrasound" and "screening", to identify relevant published carotid screening studies which have used ultrasound as a screening tool. The search strategy is shown in the flow sheet (see additional file 1).

## Results

6 studies were identified and included for overview (see additional file 2). Ballard et al[2] suggested the effectiveness of community-based programmes in early vascular disease detection. They screened 1,719 patients by carotid ultrasound. The majority of carotid screens were normal; however, 28.9% (497 patients) had 15–40% stenosis, 1.4% (24 patients) had 40–60% stenosis, and 0.3% (six patients) had >60% stenosis. The benefit of screening on a large scale successively narrowed the group of patients that were susceptible to developing disease and led to many patients being referred for further testing and risk reduction programs before they developed significant disease.

The study by Aboyans et al[3] explored the fast track method of screening carotid lesions by using a hand held ultrasound scanner which efficiently and accurately predicted the development of carotid stenosis by pre-selecting patients with atherosclerotic disease history or high risk factors. The hand held scanner detected carotid stenosis of 60% in 6% of cases with relative 100% sensitivity, and 64% specificity. Similarly, the screening by non-invasive methods, such as carotid ultrasonography accurately predicted the re-occurrence of cardiac stenosis and stroke in post-operative patients who had screening by carotid ultrasonography.

The study by D'Agostino et al[4], gathered clinical data for pre-operative, intra-operative, and post-operative patients undergoing first time isolated coronary bypass grafting. The significant predictors of possible cardiac stenosis included age, diabetes, female sex, left main coronary stenosis (>60%), prior stroke or TIA, peripheral vascular disease (PVD), post infarction angina pectoris and atrial fibrillation, carotid stenosis >50%, cardiopulmonary by-pass time, significant atherosclerosis etc. The study emphasizes the use of carotid ultrasonography screening as valuable in identifying the risk of post-operative stroke in patients and also considers it to be a diagnostic tool in patients preparing for coronary artery bypass grafting. Pre-operative carotid artery screening by carotid duplex ultrasonography in elderly patients undergoing cardiac surgery identified multiple variables as significant predictors of 80% or greater more stenosis. Most factors included female sex, PVD, history of TIA, smoking, and left main coronary artery disease where, the presence of at least one of these factors predicted 95% of the patients with 80% or greater stenosis and 91% of the patients with 50% or greater stenosis risk before operation. Thus, the probability of carotid disease was estimated to be in the range of 5%–65%. This resulted in cardiac endarterectomy with cardiac surgical procedure being performed on symptomatic patients.

Similarly, the asymptomatic carotid atherosclerosis study by Moneta et al[5] blindly compared internal carotid artery stenosis (ICAs) detected by angiography in comparison to those detected by duplex scanning. Overall the study finds accurate duplex scanning of positive predictive value (PPV) for detecting ICAs, thus proving the efficacy of carotid artery screening and decreasing unnecessary intervention. Similarly, patients with presence of systolic bruit or high arterial pressure underwent ultrasonography, where the diagnosis was further confirmed by angiography and corrective surgery protocol evaluated non-invasively.

The doppler ultrasound study by Jotkowitz et al [6] screened 124 patients where 4% tested positive for significant carotid artery stenosis. The study explored the patient's awareness particularly the significance of positive/negative test and the active role of family physician. The study indicated 82–94% of subjects were not suggested screening tests, and 59% voluntarily learned about this screening program through newspaper advertisement and similarly, were at risk of surgery that tested positive.

## Conclusion

There is great need and demand for such early screening programs, as many new patients enter the health care system. This particular screening positively contributes to the public health awareness and promotion which in the long term could potentially benefit in disease prevention, and essentially promote higher life standard for patients. It has been found that the carotid ultrasound is a non-invasive and valuable tool for detecting stenosis in patients with high risk of developing stroke.

The high specificity and sensitivity of the ultrasound means high precision in predicting the presence of disease and has been equivalently effective when evaluated against gold standard procedures including angiography which is considerably more invasive and more accurate. The common risk factors which increase the prevalence of carotid stenosis include age, female sex, previous TIA, PVD, hypercholesteremia, hypertension and smoking. The presence of any of these factors should lower the threshold for getting carotid ultrasound for such patients. It is found that the primary care plays a pivotal role in patient information, monitoring and assessment of these risk factors and symptoms. There is a higher need for General Practitioners to actively inform their patients in the benefits of this screening method and the possible advantages of non-invasive techniques, higher accuracy and efficiency of these tests. The promotion of using carotid USS is fundamental to decreasing the disease burden from hospitals and tertiary care units and moving towards more community based approach of disease prevention.

## Conflict of interests

The authors declare that they have no competing interests.

## Authors' contributions

All authors have been involved in writing the manuscript and have approved it in its final submitted form.

## Supplementary Material

Additional file 1 Click here for file

Additional file 2 Click here for file
