# Supplementary material for: Role of carotid duplex imaging in carotid screening programmes – an overview
Source: Cardiovasc Ultrasound. 2008 Jul 4;6:34. doi: 10.1186/1476-7120-6-34 (PMC2474588; doi:10.1186/1476-7120-6-34)
Supplement: Additional file 1 [file 1476-7120-6-34-S1.doc]

**Search terms:**

1. Carotid disease + screening
2. Carotid + screening
3. Carotid artery + screening
4. Carotid artery stenosis + screening
5. Carotid artery disease + screening

**Assessed for eligibility**

1. Carotid disease + screening (n=4)
2. Carotid + screening (n=14)
3. Carotid artery + screening (n=9)
4. Carotid artery stenosis + screening (n=2)
5. Carotid artery disease + screening (n=2)

N=31.

Excluded from analysis=25.

Reason= 1. Repetition of same articles by using different search criteria.

2. Other articles were not specific to the purpose of the study to

analyse the non invasice techniques of exploring carotid

techniques.
