# Supplementary material for: Role of carotid duplex imaging in carotid screening programmes – an overview
Source: Cardiovasc Ultrasound. 2008 Jul 4;6:34. doi: 10.1186/1476-7120-6-34 (PMC2474588; doi:10.1186/1476-7120-6-34)
Supplement: Additional file 2 [file 1476-7120-6-34-S2.doc]

| Study 1.Jacobwitz et al. | *Population and Screening Methods*  n=394 patients  38 (9.6%) patients with history of unilateral or bilateral CS of >50%  Rapid Duplex scan to evaluate patients for occult carotid stenosis. | *Risk factors*  1.Smoking  2.Hypertension  3.Cardiac disease    4. Hypercholesteremia | | *Risk factors and Incidence of cardiac stenosis (ICS)*  *Absence of all risk factors*🡪 Decreased ICS to 1.8%  *Presence of any one risk factor*🡪 Increased ICS 5.8% (P<0.01)  *Presence of any two risk factors*🡪ICS 13.5% (P<0.01)  *Presence of any three risk factors*🡪ICS 16.7% (P<.05)  All four risk factors🡪 ICS | *Analysis*  The prevalence of CS significantly  increases with the presence  of risk factors. |  |
| --- | --- | --- | --- | --- | --- | --- |
| 2. Ballard et al. | n=1,719 patients  Females=64% (1,094)  Males=36% (652)  Quick carotid US study. |  | | Majority screening normal.  497(28.9%) patients🡪ICS 15-40%  24(1.4%)🡪ICS 40-60%  6(0.3%) patients 🡪ICS 60% | 2 patients  got urgent  repair for  aortic aneurysms.  Non-selective vascular  screening programme  involved many patients interests.  Recommended as a key for  disease detection, prevention,  progression, and attaining  patient’s satisfaction over  health care system |  |
| 3.Abyons et al | N=197 patients  Mean age=+67 years.  Screened with fast track hand held USS and compared with carotid duplex as a gold standard. |  | | 13 (6%) cases were detected with CS.  The use of concomitant Doppler imaging through duplex did not improve accuracy.  Sensitivity of hand USS=100%  Specificity=64% | The use of fast track hand USS  can significantly predict the  risk of carotid stenosis and  unnecessary carotid duplex. |  |
| 4.D’Agostino et al | Pre, intra and post op data for  N=1, 835 patients  undergoing first time isolated coronary artery bypass grafting (CABG).  Mean age=65.3 years  N=1,279 had carotid USS. 9.3% with H/O permanent stroke or TIA  45(2.5%) patients had transient or permanent post-op neurologic event. | *Clinical risk factors predict significant carotid stenosis.*  1.Age (p<0.0001)  2.Diabetes (p=0.0123)  3.Female sex (p=0.0026)  4.Left main coronary stenosis >60% (p<0.0001)  5.Prior stroke or TIA (p=0.0008)  6.PVD (p=0.0001)  7.Prior vascular operation (p=0.0068)  8.Smoking (p<0.0001). | | *Screening for independent risk factors that predict post-op stroke*  1. Prior stroke/ TIA (p<0.0001).  2.PVD(p=0.0037)  3.Post-infarction  angina pectoris (p= 0.0319)  2. Post-operative AF (p=0.0014)  3.Carotid stenosis >50% (p=0.0029)  4. Cardiopulmonary bypass time (p=0.0006)  5.Significant atherosclerosis (p=0.0054)  6.Post-op amrinone or epinephrine use (p=0.0054)  7. Left ventricular ejection fraction <0.30 (p=0.0744) | The carotid USS identified  patients at high risk of  developing post-op stroke,  independent of any other  variables.  It is recommended to selectively  use carotid USS for patients  undergoing CABG, especially with those of prior history of  PVD or any other significant  Neurologic disease |  |
| 5. Moneta et al. | N=352 Internal carotid artery stenosis (ICAs) detected by angiography blindly compared with those detected by duplex scanning.  Duplex criteria for scanning with highest overall accuracy of  > 60% or greater stenosis and > 95% PPV. | Stroke risk in patients reduced by carotid endarterectomy with detected >60%ICAs.  Increased number of duplex scanning required for detection of ICAs. | | 90% accuracy achieved for detection of >60% stenosis. | The use of carotid duplex  Scanning accurately detects  the risk of  internal carotid stenosis  in patients undergoing  carotid endarctectomy.  It is useful for  carotid screening, and  decreases the  need for unnecessary and  invasive interventions. |  |
| 6.Jotkowitz et al. | N=124 patients underwent carotid doppler USS. |  | 5(4%) cases tested positive for significant CAS.  62% (95% CI:53-71%) learned programme through newspapers  88%(CI:82-94%) of subjects suggested GP did not suggest the test.  59% (95% CI:50-68%) did not know that the carotid artery surgery is recommended for patient who are tested positive. | | The screening method detected  CAS in voluntary group of  Patients whom learned about the  Programme from other resources  Other than primary care  GP. The unawareness of the  diagnosis was not understood in  more than half of the  patients further leading to delay  in further referrals and treatments. | |
